# The Proposed Legislation in Favour of Nursing Reform

**Published:** 1907-04-13

**Authors:** 


### Nursing Administration.

### THE PROPOSED LEGISLATION IN FAVOUR OF NURSING REFORM.

A Bill has several times been introduced into Parliament, "To regulate the qualifications of trained nurses and to provide for their registration." In view of the fact that nursing reform is the subject of all others most absorbingly interesting to nurses, it is noteworthy that the fate of this Bill, which is always a foregone conclusion, fails to excite more than the merest ripple of curiosity in their minds. An examination into its provisions may serve to explain this attitude of indifference on the part of those with whose interests it is concerned. The basis of all sound nursing politics lies in the training schools. The influence of her training school is overpowering and unique in the life of the nurse. Anything which tended to weaken this sentiment of loyalty to the traditions under which the nurse's professional training has been received would be a source of danger to nursing as a whole, and any attempt to destroy it altogether would be a futile task. Under the modern system of training the influence of the superintendent of nurses runs through the hospital. What the matron holds to be wise and wholesome in nursing matters, that is the doctrine which filters down through the sisters till it reaches the youngest probationer. The strength of this almost impalpable "prejudice" in favour of the tenets of the old training school is rendered practically indestructible when the nurse passes out of training by the extent to which her living and reputation are based on the certificate of the hospital. All the claims for consideration which a nurse possesses are based on the value of her original training. She is proud all her life of being associated with a good training school. It is her passport to employment and consideration all the world over. It brings her into immediate relations of the most cordial description with all other nurses owning the same *alma mater*, and with the medical men who have been attached to the school. Remembering these facts, and remembering, too, that the nurse, on the conclusion of her training, steps back into a world from which she finds herself differentiated by a complete shifting of outlook, and it becomes apparent that the general feeling of nurses will reflect pretty completely the feeling which prevails in the training schools. That is the great self-governing principle of the nursing profession at the present day. Loyalty to the training she has received is the central fact which regulates the precepts and practice of nurses in the absence of any State regulations.

What does the Bill to which we have alluded offer in exchange? It proposes to put the future of nursing in this country into the hands of a General Nursing Council. The Council is to have powers of a fairly complete character, except that, charged with regulating all other details of training, the length of time prescribed for the duration of the course is taken out of their hands. This is to be three years, neither more nor less, and the time is all to be spent in one institution. The Council, omnipotent in all else that concerns training, is to have its hands tied from the commencement in this important particular, although it might seem that if this were such a vital point members of a General Nursing Council could have no two opinions about it. It is impossible to make even a guess as to what the future policy of such a council would be. Hence the general disinclination of the nursing world to place themselves under its jurisdiction cannot be grounded on distaste to any particular line of policy. It is the proposed constitution of the Council, which has deprived it of any semblance of vitality, and has rendered it so transparently inefficacious that there has ceased to be any need to oppose it.

The General Nursing Council is to consist of 19 persons. Three of these are to be appointed by the Privy Council, and these are to be representatives of training schools in England and Wales, Scotland and Ireland. It is a common practice under similar circumstances for the Privy Council to appoint, in the first instance, prominent supporters of the Bill, as presumably best fitted to undertake the carrying out of its provisions. Next there are to be five medical men, one appointed by the General Council itself, so soon as it shall be constituted, three to be appointed by the British Medical Association, and one by the Medico-Psychological Association. Lastly, there are to be 11 nurses, of whom six at least are to be past or present matrons, with one asylum matron, and these will have a permanent majority in electing the President and in deciding the policy of the Council. These nurses and matrons are to be elected by the nurses on the register, and are to be voted for in the proportion of six delegates for England and Wales, two for Scotland, and two for Ireland. The mental nurses are to elect their own matron. Nothing more delusive than the process of election by popular vote in the case of such an assembly could well be devised. The only course it is possible to adopt is the circulation of a list containing certain names to those persons qualified to record their votes, with the request that the voting paper may be returned signed if the names are approved, or that some of the names may be crossed out and others substituted if the voter wishes to secure the election of any one else. It is hardly too much to say that an instance has never been known in which the votes of individuals have had the smallest effect in influencing the appointment of a council under such conditions. The proceeding resolves itself into a vote of confidence in the policy of the Council, but it is one which cannot rouse the smallest enthusiasm even in the most inexperienced voter. A council thus elected is in reality appointed by itself, for the committee charged with the duty of preparing the list, can hardly fail to regard the supporters of its own policy as the candidates most proper to be selected. It is significant that in the Provisional Council which is to be charged with making arrangements for the election of the first General Nursing Council, four seats out of eleven are to be at the disposition of the twin societies promoting the Bill.

There is no way in which popular election can be made effectual for the appointment of a council able to deal with highly intricate and technical details in a broad and statesmanlike manner. It is not merely a question of having opinions, it is a question of knowing the right men and women, of being behind the scenes and well acquainted with the principles and practice of the great training schools as a whole. And this is knowledge which is not accessible to the great body of nurses, and never from the nature of the case could it be accessible to them. We believe that their instinct in these matters to be guided by their training school is wise and sincere, and we are confident that until the question of regulating the nursing profession is seriously taken in hand by the training schools as a cohering body, nurses throughout the kingdom will continue to regard nursing legislation with indifference. It is quite clear that they are not greatly tempted by the prospect of possessing such shadowy powers as under this ill-starred Bill it is proposed to put in their hands.

